# Glyoxalase 1 Confers Susceptibility to Schizophrenia: From Genetic Variants to Phenotypes of Neural Function

**DOI:** 10.3389/fnmol.2021.739526

**Published:** 2021-11-01

**Authors:** Jingwen Yin, Guoda Ma, Shucun Luo, Xudong Luo, Bin He, Chunmei Liang, Xiang Zuo, Xusan Xu, Qing Chen, Susu Xiong, Zhi Tan, Jiawu Fu, Dong Lv, Zhun Dai, Xia Wen, Dongjian Zhu, Xiaoqing Ye, Zhixiong Lin, Juda Lin, You Li, Wubiao Chen, Zebin Luo, Keshen Li, Yajun Wang

**Affiliations:** ^1^Department of Psychiatry, Affiliated Hospital of Guangdong Medical University, Zhanjiang, China; ^2^Institute of Neurology, Guangdong Medical University, Zhanjiang, China; ^3^Department of Radiology, Affiliated Hospital of Guangdong Medical University, Zhanjiang, China; ^4^Maternal and Children's Health Research Institute, Shunde Maternal and Children's Hospital, Guangdong Medical University, Foshan, China; ^5^Center for Cognitive and Brain Sciences, Institute of Collaborative Innovation, University of Macau, Macao SAR, China; ^6^Department of Psychology, Faculty of Social Sciences, University of Macau, Macao SAR, China; ^7^Department of Neurology and Stroke Center, The First Affiliated Hospital, Jinan University, Guangzhou, China; ^8^Clinical Neuroscience Institute, Jinan University, Guangzhou, China

**Keywords:** schizophrenia, Glo-1, rs1781735, eQTL, mRNA expression, promoter activity, enzymatic activity, brain function

## Abstract

This research aimed to investigate the role of glyoxalase 1 (Glo-1) polymorphisms in the susceptibility of schizophrenia. Using the real-time polymerase chain reaction (PCR) and spectrophotometric assays technology, significant differences in Glo-1 messenger ribonucleic acid (mRNA) expression (*P* = 3.98 × 10^−5^) and enzymatic activity (*P* = 1.40 × 10^−6^) were found in peripheral blood of first-onset antipsychotic-naïve patients with schizophrenia and controls. The following receiver operating characteristic (ROC) curves analysis showed that Glo-1 could predict the schizophrenia risk (*P* = 4.75 × 10^−6^ in mRNA, *P* = 1.43 × 10^−7^ in enzymatic activity, respectively). To identify the genetic source of Glo-1 risk in schizophrenia, Glo-1 polymorphisms (rs1781735, rs1130534, rs4746, and rs9470916) were genotyped with SNaPshot technology in 1,069 patients with schizophrenia and 1,023 healthy individuals. Then, the impact of risk polymorphism on the promoter activity, mRNA expression, and enzymatic activity was analyzed. The results revealed significant differences in the distributions of genotype (*P* = 0.020, false discovery rate (FDR) correction) and allele (*P* = 0.020, FDR correction) in rs1781735, in which G > T mutation significantly showed reduction in the promoter activity (*P* = 0.016), mRNA expression, and enzymatic activity *(P* = 0.001 and *P* = 0.015, respectively, GG vs. TT, in peripheral blood of patients with schizophrenia) of Glo-1. The expression quantitative trait locus (eQTL) findings were followed up with the resting-state functional magnetic resonance imaging (fMRI) analysis. The TT genotype of rs1781735, associated with lower RNA expression in the brain (*P* < 0.05), showed decreased neuronal activation in the left middle frontal gyrus in schizophrenia (*P* < 0.001). In aggregate, this study for the first time demonstrates how the genetic and biochemical basis of Glo-1 polymorphism culminates in the brain function changes associated with increased schizophrenia risk. Thus, establishing a combination of multiple levels of changes ranging from genetic variants, transcription, protein function, and brain function changes is a better predictor of schizophrenia risk.

## Introduction

Schizophrenia is a devastating and complex neurodevelopmental disorder with highly heterogeneous and multifaceted clinical manifestations (Cannon, 1996; Allen et al., 2008). It has been postulated that a combination of multiple environmental and genetic predisposing factors may confer risk for schizophrenia (Yin et al., 2014; Modai and Shomron, 2016). The variants in risk genes might lead to dysregulation of molecular pathways and aberrations in the structure and function of the brain, which could be reflected by the abnormalities in cognitive function, emotions, and behaviors (Owen et al., 2016; Klingler et al., 2021). To systematically understand how genes confer risks across these levels of schizophrenia will help to build a spectrum of abnormal states, predict vulnerable processes, design personalized diagnostic, and therapeutic tools (Klingler et al., 2021).

Carbonyl stress is an abnormal metabolic state that results in the accumulation of dicarbonyl compounds, such as methylglyoxal (MG) and glyoxal (Miyata et al., 1999) leading to the eventual formation of advanced glycation end products (AGEs). The accumulation of MG and AGEs ultimately induces severe cytotoxicity in the neurons and causes dysfunction of the nervous system. The recent studies in animals and humans have demonstrated that enhanced carbonyl stress contributes to the onset of various neurological disorders, such as schizophrenia (Itokawa et al., 2018; Yoshioka et al., 2021), Parkinson's disease (Kurz et al., 2011), Alzheimer's disease (Kuhla et al., 2007; Frandsen et al., 2020; Lv et al., 2020), mood disorder (Fujimoto et al., 2008), autism (Junaid et al., 2004; Gabriele et al., 2014; Kovac et al., 2015), panic disorder (Politi et al., 2006), and anxiety behavior (Hovatta et al., 2005; Distler et al., 2012; Du et al., 2019).

Glyoxalase 1 (Glo-1), a rate-limiting enzyme in the glyoxalase system, metabolizes the MG into S-D-lactoylglutathione and effectively decreases the formation of AGEs (He et al., 2020; Sarker et al., 2020). In contrast, the impairment of Glo-1 leads to carbonyl stress and, eventually, the accumulation of AGEs in the brain (Wang et al., 2019; Kold-Christensen and Johannsen, 2020). Therefore, Glo-1 might play a key role in the balance of cellular detoxification and toxification in reactive carbonyl species (Hara et al., 2021). This process has been reported as a possible causative factor in the etiology of schizophrenia (Mizutani et al., 2019; Ohnishi et al., 2019).

The Glo-1 gene is located at human chromosome 6p21.2, which has previously been reported as an associated region for schizophrenia in multicenter genome-wide association studies (GWAS) conducted on large samples (International Schizophrenia et al., 2009; Shi et al., 2009; Stefansson et al., 2009; Schizophrenia Working Group of the Psychiatric Genomics, 2014). In particular, Glo-1 has been identified as a target gene of miR-137 (Lv et al., 2018), which is strongly associated with schizophrenia, as reported by GWAS (Schizophrenia Psychiatric Genome-Wide Association Study (GWAS) Consortium, 2011; Ma et al., 2014; Wang et al., 2020). Reduced expression of Glo-1 and, thus, increased accumulation of glycated dicarbonyl compounds have been reported in a subclass of schizophrenia (Kouidrat et al., 2013; Katsuta et al., 2014). The Glo-1 mutation caused structural alteration of the neurites in the post-mortem human cerebral tissues of patients with schizophrenia (Mizutani et al., 2019). Furthermore, a missense substitution (Glu111Ala) and heterozygous frameshift mutations were recently shown to significantly decrease the Glo-1 protein expression and enzymatic activity in the red blood cells (RBCs) from Japanese patients with schizophrenia (Arai et al., 2010; Miyashita et al., 2014a,b; Torii et al., 2020).

Given the critical role of Glo-1 in the detoxification of carbonyl stress, we sought to test the hypothesis that Glo-1 is involved in the pathogenesis of schizophrenia. The case-control studies were performed to identify Glo-1 genetic variants, mRNA expression, and enzymatic activity in the Han population of China. To examine the effects of Glo-1 on phenotypes of brain function in schizophrenia, an effective proxy of Glo-1 expression was selected, and neural imaging analysis was performed using the functional magnetic resonance imaging (fMRI) combined with the Glo-1 brain expression pattern. Our investigation improved the understanding of the effect of Glo-1 on schizophrenia and provides a referable analysis workflow in imaging genetics research.

## Subjects And Methods

### Subjects and Clinical Assessment

This hospital-based case-control study consecutively recruited 1,069 patients with schizophrenia (684 men and 385 women) from the Department of Psychiatry at the Affiliated Hospital of Guangdong Medical University, Guangdong Province, China. All the subjects were Han Chinese from the local area of southern China, received a structured clinical interview and were independently diagnosed by the two experienced senior psychiatrists according to the criteria of the Diagnostic and Statistical Manual of Mental Disorders, Fifth Edition (DSM-5). The patients were excluded from the study if they had any physical disease, especially renal dysfunction or diabetes mellitus (because these diseases may affect Glo-1 expression). The Positive and Negative Syndrome Scale (PANSS) was used to evaluate the psychotic symptoms in the patients with schizophrenia. The control group consisted of 1,023 healthy individuals (619 men and 404 women) who were recruited from the same geographical area as the patients and had no known history of major psychiatric disorders, serious physical disease, or substance abuse. For the imaging-genetics subgroup, 91 first-onset antipsychotic-naïve patients with schizophrenia who completed all the imaging and genetics protocols were recruited. All the participants were right-handed. For the biochemical analysis, first-onset antipsychotic-naïve patients with schizophrenia and age- and sex-matched controls were recruited. There were 63 patients and 53 controls for mRNA expression analysis and 74 patients and 73 controls for enzymatic activity analysis. The study protocol was reviewed and approved by the ethics committee of the Affiliated Hospital of Guangdong Medical University (IRB number, PJ2017058). All the subjects or their relatives provided informed consent to participate in the study.

### Measurement of Glo-1 Enzymatic Activity and mRNA Expression

For mRNA analysis, fresh blood samples were obtained, and total RNA was extracted from the peripheral blood using the PaxGene Blood RNA Kit (Sangon, Shanghai, China). A real-time PCR was performed using the SYBR Premix Ex Taq kit (Takara, Dalian, China) according to the instructions from the manufacturer using the primers listed in Supplementary Table 1. Glyceraldehyde-3-phosphate dehydrogenase (GAPDH) was used as the endogenous control, and the comparative cycle threshold (Ct) method was used to quantify the Glo-1 mRNA levels. Each reaction was performed three times. For enzymatic activity analysis, the RBCs were separated from the venous blood samples by centrifugation (2,000 g, 5 min). The Glo-1 enzymatic activity was determined with a spectrophotometer at 240 nm by measuring the formation of S-D-lactoylglutathione as reported in a previous study by Gabriele et al. (2014).

### Single Nucleotide Polymorphism (SNP) Selection

**Figure 1:**
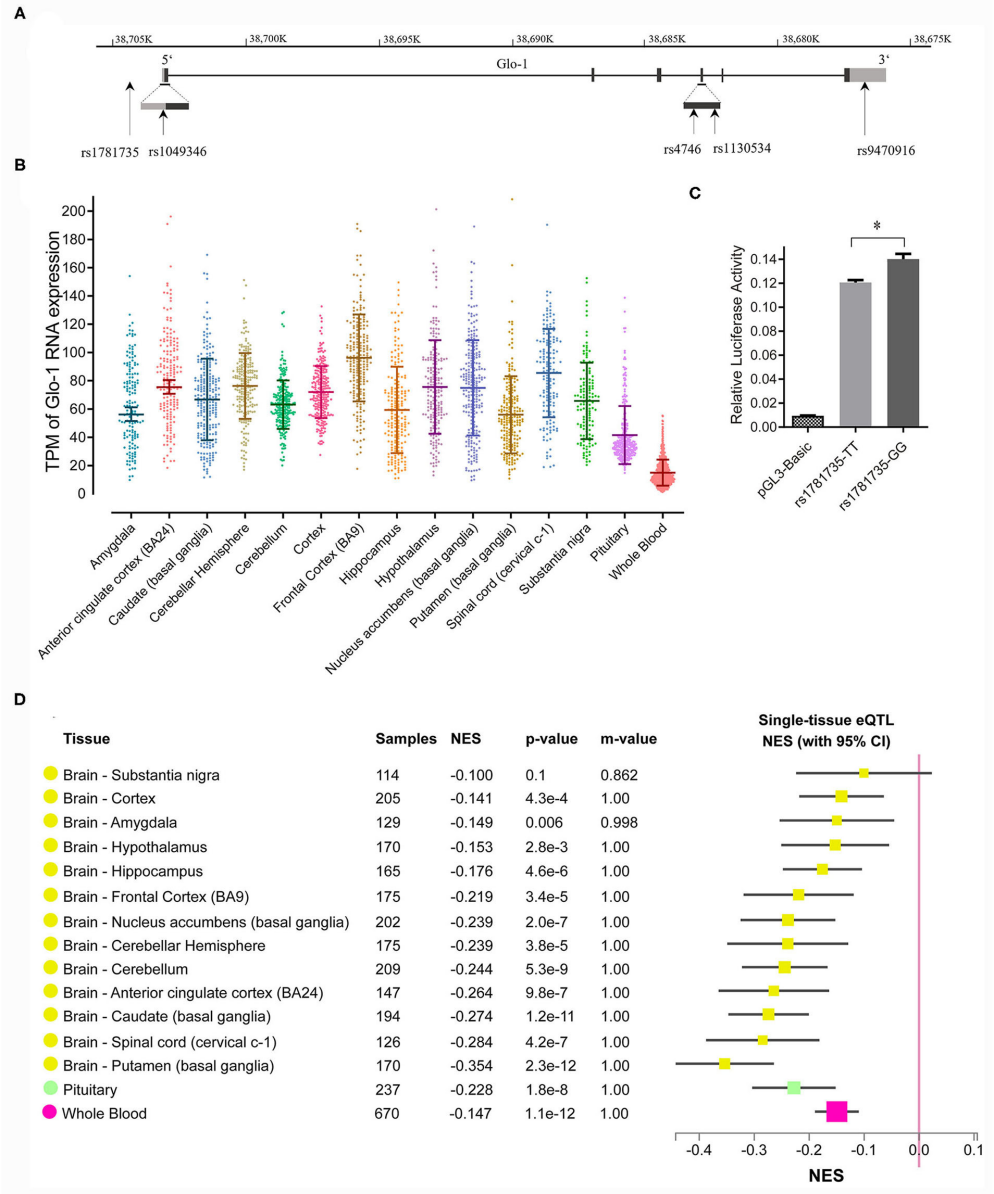
**(A)** Distribution of single nucleotide polymorphisms (SNPs) across the Glo-1 gene selected for the association analysis. Exons are shown as black boxes (exons), and intron sequences are shown as lines. The gray boxes represent the 3' untranslated region (3'-UTR) and 5' untranslated region (5'-UTR). The vertical lines indicate the locations of these polymorphisms. Five SNPs were identified in the Glo-1 gene from the HapMap/Haploview database and the literature; **(B)** transcripts per kilobase million (TPM) values of the Glo-1 expression pattern in the brain tissues and whole blood in healthy participants. **(C)** Effect of the rs1781735 T/G polymorphism on the transcriptional activity of the Glo-1 promoter. *, *P* < 0.05. The error bar in the bar plot represents the SD of the mean. **(D)** GTEx Multitissue expression quantitative trait locus (eQTL) Plot for rs1781735. NES, normalized effect size. *P*-value, from a *t*-test that compares the observed NES from single-tissue eQTL analysis to a null NES of 0; m-value, the posterior probability that an eQTL effect exists in each tissue tested in the cross-tissue meta-analysis. Small m-value (e.g., <0.1): the study is predicted to not have an effect. Large m-value (e.g., >0.9): the study is predicted to have an effect.

The criteria for identifying SNPs included a linkage disequilibrium (LD) r^2^ value >0.8 and a minor allele frequency (MAF) of more than 0.10 in the Han population of China. Five candidate SNPs (rs1781735, rs1049346, rs1130534, rs4746, and rs9470916) were selected based on the data from the 1,000 Genomes Project and the most reported SNPs (Gale et al., 2004b; Barua et al., 2011; Peculis et al., 2013; Gabriele et al., 2014; Tao et al., 2016). Therefore, these loci were used for the subsequent analysis (detailed information is shown in Figure 1A). The SNP Rs4746 (also called rs2736654), in exon 4 of the Glo-1 gene, is a C > A change that causes Ala111Glu and is associated with a decrease in Glo-1 enzymatic activity (Barua et al., 2011). In addition, T > A in rs1130534 causes a synonymous substitution at codon 124 (GGA to GGT and Gly to Gly). It was reported that the reduced MG concentrations in human whole blood cell lysates correlated with the A allele (Peculis et al., 2013), which may represent a marker for susceptibility to Glo-1-related diseases (Gabriele et al., 2014; Tao et al., 2016). The SNP rs9470916 is located in the 3′-untranslated region of the Glo-1 gene. The variant rs1781735 is located in the promoter and has complete linkage with rs1049346 (c.-7C>T) which exerts significant effects on Glo-1 transcriptional activity (Gale et al., 2004a). Therefore, we genotyped the rs1781735 SNP, which can serve as a proxy for rs1049346.

### Genotyping

A human genomic DNA was isolated from the EDTA-treated peripheral blood samples using a commercially available kit (Tiangen Biotech, Beijing, China) according to the instructions from the manufacturer. The selected Glo-1 SNPs were genotyped using the SNaPshot technique as described in the previous studies (Ma et al., 2014; Fu et al., 2017), and the primers are listed in Supplementary Table 2.

### Luciferase Reporter Assay

The DNA fragments of the human Glo-1 promoter containing either the T allele or the G allele at rs1781735 were cloned into the pGL3 vector, and the promoter activities were determined in SH-SY5Y cells. The Glo-1 promoter fragments encompassing nucleotides from −1,200 base pairs (bp) to +22 bp (relative to transcription start site +1) were amplified, which came from the two individual homozygotes with respect to the corresponding genotypes (GG and TT) for rs1781735. Next, we cloned the amplified fragments into the pGL3-basic plasmid vectors using the primers shown in Supplementary Table 3 and verified the constructs using bidirectional DNA sequencing. The reporter constructs containing either the G allele or the T allele were transiently cotransfected into SH-SY5Y cells along with the *Renilla* reporter gene (pRLTK) for internal normalization. After a 48-h incubation, the luciferase and *Renilla* luciferase activities were measured using the Dual-Luciferase Reporter Assay System according to the instructions from the manufacturer (Promega Corporation, Madison, WI, USA).

### Gene Expression Patterns and Expression Quantitative Trait Locus (eQTL) Analysis

We replicated Glo-1 expression levels and the eQTL of the G/T polymorphism at rs1781735 using data from Genotype-Tissue Expression (GTEx; https://www.gtexportal.org) Analysis Release V8 (dbGaP: phs000424.v8.p2) in 14 brain regions (Sul et al., 2013; Human Genomics, 2015; Melé et al., 2015).

### fMRI Acquisition and Data Processing

The resting-state fMRI images and high-resolution T1-weighted images were acquired using a 3.0-T GE (Discovery MR750) Signa Scanner (GE Medical Systems, WI, USA) with an 8-channel phased-array head coil at the Department of Radiology of Affiliated Hospital of Guangdong Medical University. The high-resolution T1-weighted images were obtained using three-dimensional T1-weighted magnetization-prepared rapid gradient echoes (MPRAGE) with the following parameters: time of repetition (TR) = 8.16 ms, time of echo (TE) = 3.18 ms, field of view (FOV) = 512 mm × 512 mm, flip angle = 9 degrees, slice thickness/gap = 1 mm, matrix = 256 × 256, slices = 172. The fMRI images were collected using a gradient-echo planar imaging (EPI) sequence. The imaging parameters were as follows: TR = 2,000 ms, TE = 30 ms, FOV = 230 mm × 230 mm, matrix = 64 × 64, slices = 38, slice thickness = 3.6 mm, 240 dynamics, and scan time = 8 min.

The amplitude of low-frequency fluctuation (ALFF) based on the blood oxygen level-dependent (BOLD) fMRI signals was analyzed. Data pre-processing was performed using Statistical Parametric Mapping (SPM) 12 software (http://www.fil.ion.ucl.ac.uk/spm/) based on MATLAB 2019. First, the initial 10 scan volumes were discarded to allow for steady-state magnetization. The slice timing and head motion were corrected using the remaining scan volumes. The motions exceeding 2 mm displacement and 2 degrees rotation were excluded. The standard Montreal Neurological Institute (MNI) EPI template (3 mm isotropic voxels) was used for spatial normalization. Spatial smoothing was performed using a Gaussian kernel of 6 × 6 × 6 mm full width at half-maximum. Finally, temporal handpass filtering (0.01–0.08 Hz) was conducted to remove the low-frequency drifts and physiological high-frequency noise.

### The Statistical Analyses

Statistical analysis was performed using SPSS 22.0 software (SPSS, Chicago, IL, USA), and statistical graphs were generated using GraphPad Prism version 8.0.2 (GraphPad Software Inc, San Diego, CA, USA). Hardy-Weinberg equilibrium (HWE) was tested using the χ^2^ test. The statistical analyses comparing the genotypes and allele distributions were performed using the χ^2^ test. The generalized odds ratios (*OR*s) and 95% confidence interval (CI) of the alleles were calculated. The multiple comparisons were performed using false discovery rate (FDR) correction. The Linkage disequilibrium (LD) status and haplotype analysis were determined using the Haploview 4.2 program. Only those haplotypes with frequencies >3% were further analyzed. The power calculations were performed using QUANTO 1.2 software. The descriptive statistics of the clinical characteristics, mRNA expression, and enzymatic activity of Glo-1 are presented as the mean ± standard deviation (SD). The statistical analyses were performed using the independent-sample *t*-tests. The receiver operating characteristic (ROC) curves were constructed from the mRNA expression and enzymatic activity data in the patients with schizophrenia. The value of *P* < 0.05 was considered statistically significant for the above tests. Transcripts per kilobase million (TPM) of RNA-sequencing and eQTL data in the GTEx database were downloaded and extracted using R version 3.6.2. (R Core Team, Vienna, Austria).

A multitissue eQTL plot for rs1781735 was calculated in the GTEx browser (Sul et al., 2013). The normalized effect size (NES) of the eQTLs is defined as the slope of the linear regression of normalized expression data vs. the three genotype categories using single tissue eQTL analysis, representing eQTL effect size. It is computed as the effect of the alternative allele (ALT) T relative to the reference allele (REF) G in rs1781735 (chr6_38704303_G_T_b38) of Glo-1 in the reference human genome GRCh38/hg38.

The second-level analysis of fMRI data was performed using the SPM 12 software. The independent-sample *t*-tests were used to examine the genotype effects on ALFF with sex, age, and education as covariates. The multiple comparison corrections were performed within each analytical imaging modality using familywise error (FWE) corrected at a two-tailed threshold of *P* < 0.05. The results were visualized by BrainNet Viewer version 1.7 (Beijing Normal University, Beijing, China) (Xia et al., 2013).

## Results

### Glo-1 Confers the Risk of Schizophrenia

**Figure 2:**
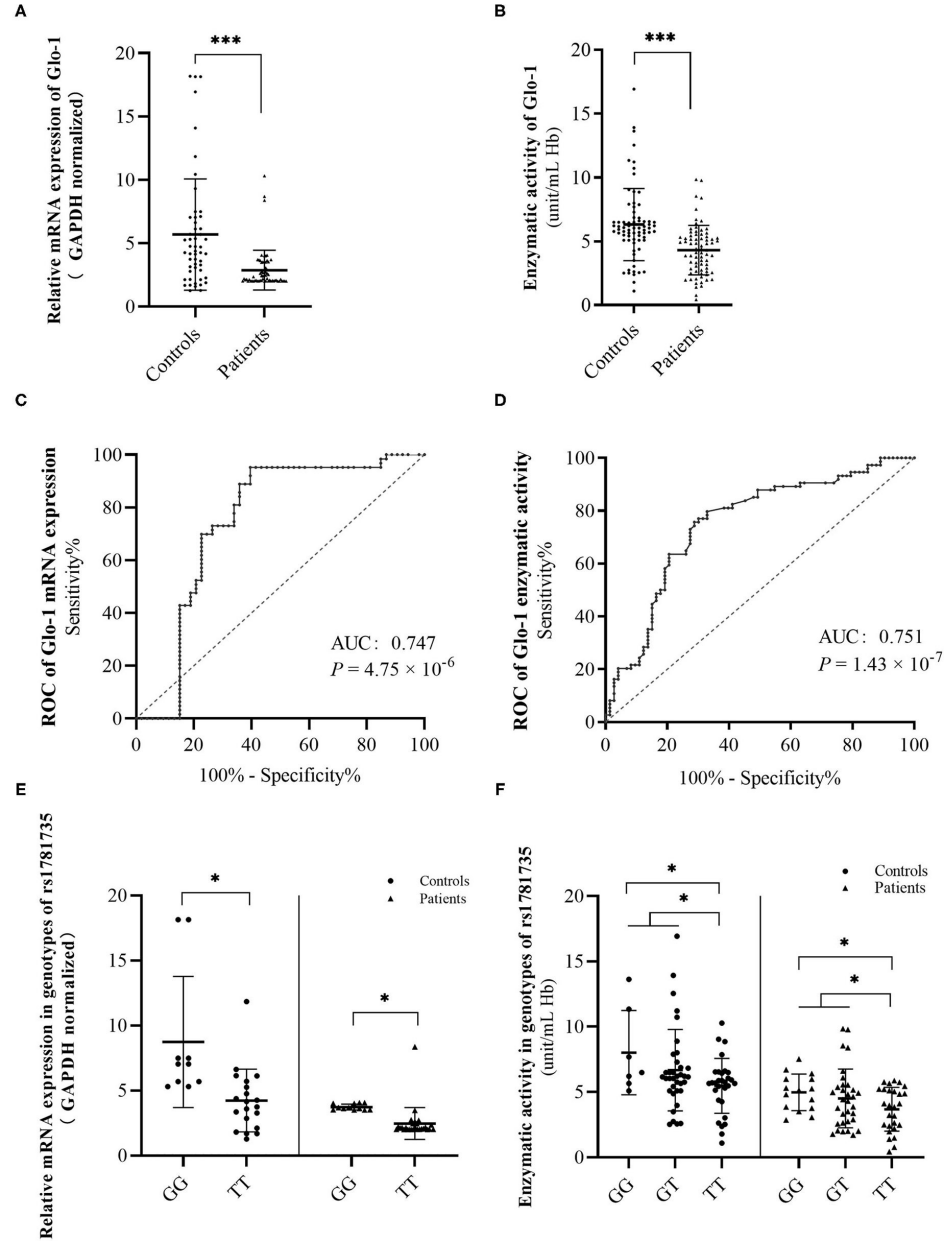
Discrimination of glyoxalase 1 (Glo-1) mRNA levels **(A)** and enzymatic activity levels **(B)** in peripheral blood from the first-onset antipsychotic-naïve patients with schizophrenia and healthy controls. A receiver operating characteristic (ROC) curve was constructed to evaluate the role of Glo-1 mRNA expression **(C)** and enzymatic activity **(D)** in the diagnosis of schizophrenia. Discrimination of Glo-1 mRNA levels **(E)** and enzymatic activity levels **(F)** in genotypes of rs1781735 in peripheral blood of first-onset antipsychotic-naïve patients with schizophrenia and healthy controls. AUC, the area under the ROC curve. *, *P* < 0.05. ***, *P* < 0.001. The error bar represents the SD of the mean.

To explore the role of Glo-1 in the risk of schizophrenia, the biochemical effect of Glo-1 was analyzed in first-onset antipsychotic-naïve patients with schizophrenia and age- and sex-matched controls (Supplementary Table 4). Both the mRNA expression and enzymatic activity were much lower in the patients with schizophrenia than in controls (*t* = 4.42, *P* =3.98 × 10^−5^; *t* = 5.02, *P* = 1.40 × 10^−6^, respectively) (Figures 2A,B and Supplementary Table 4).

The ROC curves were constructed to further evaluate the correlation of Glo-1 mRNA and enzyme activity with the diagnosis of schizophrenia. For Glo-1 mRNA, the area under the curve (AUC) was 0.747 (*P* = 4.75 × 10^−6^, 95% *CI*: 0.65–0.85), representing a potential diagnostic capability. The calculation of the cut-off value was based on the maximum Youden Index (sensitivity + specificity −1). The cut-off value of the mRNA expression level was 4.69 (ΔΔCT), with the sensitivity and specificity values of 0.952 and 0.604, respectively (Figure 2C). For Glo-1 enzyme activity, the AUC was 0.751 (*P* = 1.43 × 10^−7^), the 95% *CI* was 0.671–0.832, and the cut-off value was 3.05 (unit/ml Hb), with the sensitivity and specificity values of 0.671 and 0.797, respectively (Figure 2D). These combined results indicated that Glo-1 may confer a risk for schizophrenia.

### The rs1781735 T/G Polymorphism Might Be the Genetic Source of Schizophrenia Vulnerability

To further identify the Glo-1 genetic source of risk for schizophrenia, a case-control study was performed in which 1,069 patients with schizophrenia and 1,023 healthy controls were recruited to analyze the risk effects of Glo-1 variants on schizophrenia. The demographic characteristics of the sample are shown in Supplementary Table 5. There were no significant differences in the demographic characteristics between the patients with schizophrenia and healthy controls and all selected SNPs were in HWE (*P* > 0.05). The significant differences were observed in SNP rs1781735 between the patients with schizophrenia and healthy controls for genotype (χ^2^ = 8.64, *P* = 0.020) and allele (χ^2^ = 6.59, *P* = 0.020) distributions after FDR correction (Table 1). The T allele of rs1781735 was more frequent in the patients with schizophrenia than in the healthy controls. However, no statistically significant associations of allele or genotype distributions with schizophrenia were observed for rs9470916, rs1130914, or rs4746 (all *P* > 0.05). The haplotype analysis was conducted for these SNPs, and the corresponding results are shown in Supplementary Table 7. The frequency of the C-T-A-G haplotype, corresponding to the rs9470916-rs1130534-rs4746-rs1781735 polymorphism, was significantly lower overall in the patients with schizophrenia than in the healthy controls (χ^2^ = 5.13, *OR* = 0.86, 95% *CI* (0.75–0.98), *P* = 0.024). The LD of these SNPs is shown in Supplementary Table 6. The power calculations were performed using the MAF of each allele with an *OR* of 1.5 at the 0.05 level (Supplementary Table 8). The estimated statistical power was from 0.993 to 1. Our findings could be statistically strong with appropriate sample size.

**Table 1:** Genotype and allele distributions of glyoxalase 1 (Glo-1) polymorphisms in the participants.

**SNP**	**N**	**Genotype N (%)**			**χ^2^**	***P***	*P* ^*^	**Allele N (%)**		**χ^2^**	***P***	*P* ^*^	**OR**	**95% CI**
rs1781735		GG	GT	TT				G	T					
Patient	1,069	144 (13.47)	468 (43.77)	457 (42.75)	8.64	0.01^*^	0.020^*^	756 (35.36)	1,382 (64.63)	6.59	0.010^*^	0.020^*^	0.85	0.75–0.96
Control	1,023	152 (14.85)	498 (48.68)	373 (36.46)				802 (39.19)	1,244 (60.8)					
rs9470916		AA	CA	CC				A	C					
Patient	1,069	66 (6.17)	427 (39.94)	576 (53.88)	3.01	0.222	0.333	559 (26.14)	1,579 (73.85)	1.35	0.246	0.295	1.09	0.94–1.25
Control	1,023	66 (6.45)	371 (36.26)	586 (57.28)				503 (24.58)	1,543 (75.41)					
rs1130534		AA	AT	TT				A	T					
Patient	1,069	66 (6.17)	437 (40.87)	566 (52.94)	3.41	0.182	0.273	569 (26.61)	1,569 (73.38)	1.55	0.212	0.254	1.09	0.95–1.25
Control	1023	66(6.45)	378(36.95)	579(56.59)				510(24.92)	1536(75.07)					
rs4746		CC	CA	AA				C	A					
Patient	1,069	30 (2.8)	266 (24.88)	773 (72.31)	3.05	0.218	0.327	326 (15.24)	1,812 (84.75)	2.93	0.087	0.261	1.16	0.98–1.38
Control	1,023	20 (1.95)	234 (22.87)	769 (75.17)				274 (13.39)	1,772 (86.6)					

N, number; OR, odds ratio; 95% CI; P*, P value calculated using a false discovery rate (FDR) correction;

* *, P < 0.05*.

### The rs1781735 T/G Polymorphism Might Be a Functional SNP of Glo-1 in Schizophrenia

We speculated that the rs1781735 SNP, located in the promoter region of Glo-1, may affect Glo-1 expression and ultimately contribute to the pathogenesis of schizophrenia. To evaluate the effects of rs1781735 T/G on Glo-1 transcription activity, we cloned DNA fragments of the human Glo-1 promoter, containing either the T allele or the G allele at rs1781735, into the pGL3 vector, and the promoter activities were assessed in the SH-SY5Y cells. As shown in Figure 1C, the luciferase assay indicated that the Glo-1 promoter containing the T allele of rs1781735 reduced the relative promoter activity by 11.2% compared with the G allele in the SH-SY5Y (*P* = 0.016) cells.

Next, we suspected that rs1781735 may affect the Glo-1 mRNA expression and enzymatic activity in the patients with schizophrenia and healthy controls. The mRNA expression and enzymatic activity data were grouped by genotypes of rs1781735. The differences in mRNA expression were found in both the patients with schizophrenic (*t* = 3.54, *P* = 0.001) and controls (*t* = 3.34, *P* = 0.003) when the GG genotype was compared with the TT genotype (Figure 2E and Supplementary Table 4). For Glo-1 enzymatic activity, the differences were observed in the patients with schizophrenia and healthy controls when the GG genotype was compared to TT genotype (*t* = 2.54, *P* = 0.015 in patients, *t* = 2.59, *P* = 0.014 in controls) and when the GG + GT genotype was compared with the TT genotype (*t* = 2.13, *P* = 0.036 in patients, *t* = 2.16, *P* = 0.034 in controls) (Figure 2F and Supplementary Table 4). The T allele in rs1781735 has a decreasing tendency of mRNA expression and enzymatic activity compared with the G allele in the peripheral blood of both first-onset antipsychotic-naïve patients with schizophrenia and healthy controls.

Collectively, these data indicate that rs1781735 is likely a functional SNP, and sequence changes at this site can influence the Glo-1 transcription activation, mRNA expression, and enzymatic activity in both the patients with schizophrenia and healthy controls.

### Glo-1 Is Widely Expressed in the Brain and the rs1781735 T/G Polymorphism Could Be an eQTL for Glo-1

We further evaluated the functional relevance of the rs1781735 polymorphism in Glo-1 expression in the brain. The Glo-1 expression patterns in the brain were defined and clarified using eQTL analysis conducted in the 14 brain regions and whole blood in the post-mortem healthy participant data from the GTEx database. The Glo-1 was broadly expressed in the 14 brain regions {such as the frontal cortex [Brodmann area 9 (BA9)], cerebellar hemisphere, and anterior cingulate cortex (BA24).} and whole blood (Figure 1B). As shown in Figure 1D, rs1781735 affected the Glo-1 gene expression at the mRNA level in the 14 human brain regions and whole blood, and the TT genotype was associated with the reduced Glo-1 expression compared with the GG genotypes in the 14 human brain regions and whole blood.

### The rs1781735 T/G Polymorphism Affects the Brain Function in First-Onset Antipsychotic-Naïve Patients With Schizophrenia

We hypothesized that Glo-1 might affect the brain function of schizophrenia, considering its crucial role in neurodetoxification. To examine the functional effect of Glo-1 in the brain, rs1781735 was applied as a proxy of Glo-1 expression in the neuroimaging analysis for the Glo-1 functional localization in the brain of patients with schizophrenia.

**Figure 3:**
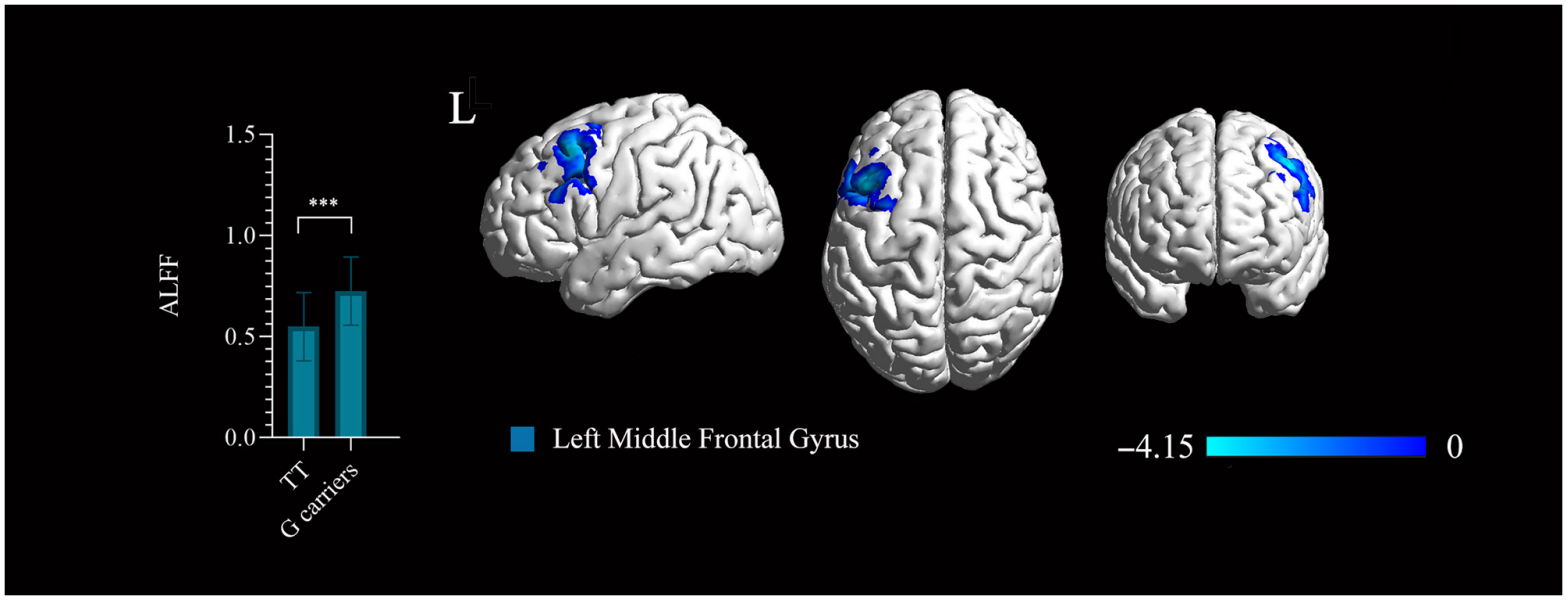
Significant regions mapped onto the standard Ch2 Template using BrainNet Viewer. The rs1781735 genotype effect on the amplitude of low-frequency fluctuation (ALFF) map in the patients (TT genotype > G carriers). Areas colored blue gradient correspond to the *t*-value. ***, *P* < 0.001, familywise error (FWE) corrected. The error bar represents the SD of the mean.

The ALFF based on BOLD fMRI signals was analyzed to evaluate the effect of Glo-1 on neuronal activation in the brain. There were no significant differences in the age, sex, years of education, symptom phenotypes evaluated by PANSS between the genotype groups (Supplementary Table 9). In first-onset antipsychotic-naïve patients with schizophrenia, TT genotype exhibited significantly decreased ALFF in the left middle frontal gyrus (BA9) (Talairach: −45, 18, 51; 236 mm^3^, *t* = 4.15, *P* < 0.001, FWE corrected) compared with the G carriers (combined GG and GT genotypes) (Figure 3).

## Discussion

This study investigated the effect of Glo-1 on schizophrenia risks at multiple levels, such as genetic variants, transcription, expression, protein function, and phenotypes.

The previous studies identified downregulated Glo-1 expression in the blood of Japanese patients with schizophrenia (Arai et al., 2010), suggesting that the dysregulation of Glo-1 might be related to the molecular causes or consequences of schizophrenia. To further identify the effect of Glo-1 on the risk of schizophrenia and more accurately measure the changes in Glo-1, we attempted to reduce or eliminate the influence of other factors, such as antipsychotic drugs and disease treatment period, on the results. To maintain the homogeneity in the cohort in this study, we analyzed Glo-1 expression and enzymatic activity in the peripheral blood of antipsychotic-naïve first-episode patients with schizophrenia and a sex-, age-, and education-matched healthy control group from the same community. We demonstrated that both the Glo-1 mRNA expression and activity were significantly decreased in the blood of patients compared with the healthy controls. These results indicate credible evidence of the potential biomarker effect of Glo-1 on schizophrenia.

**Figure 4:**
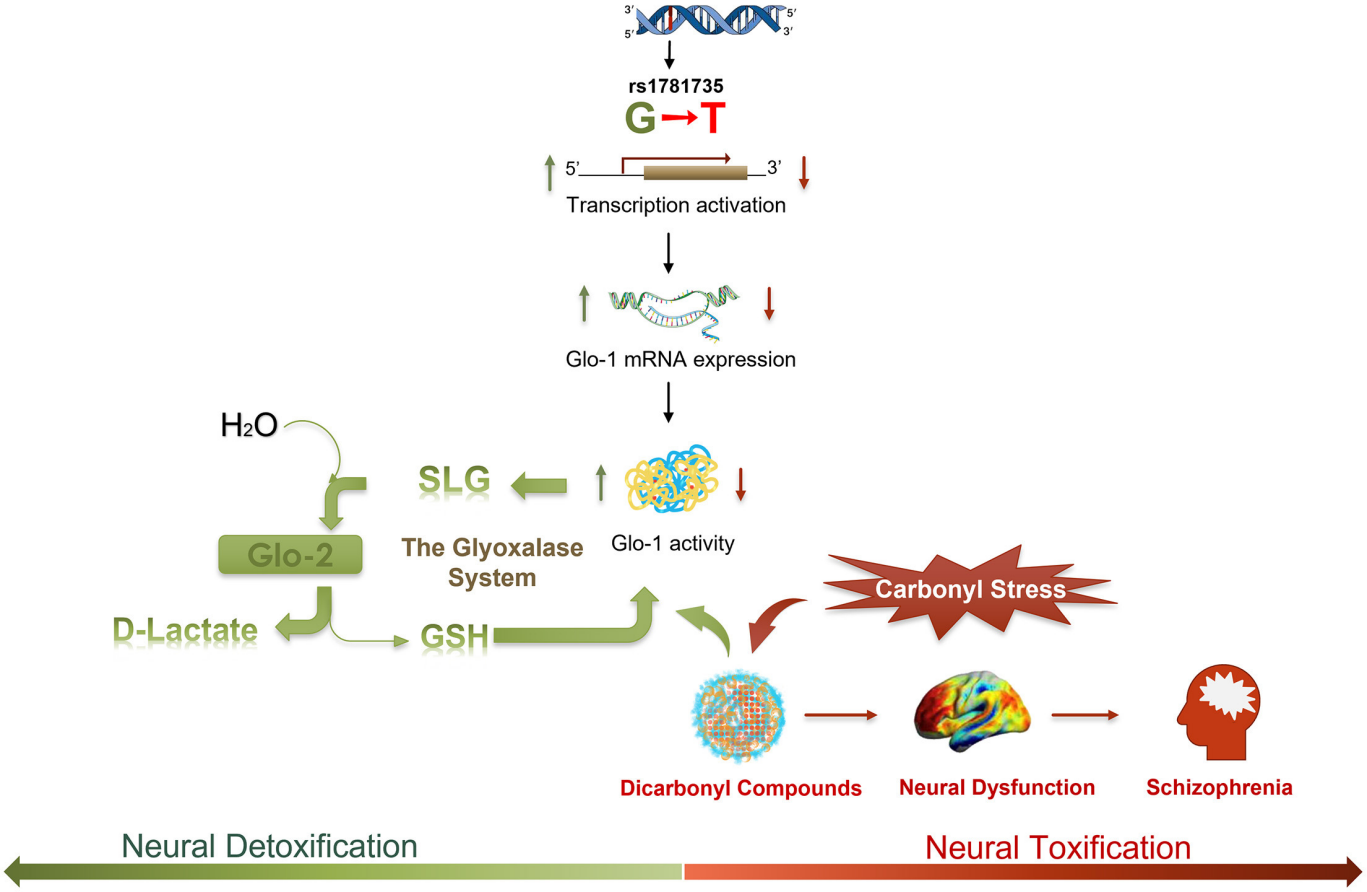
A schematic illustrating the hypothesized regulatory process of the rs1781735 G-T mutation in the etiology of schizophrenia. Dicarbonyl compounds, such as methylglyoxal (MG), as a toxic species could lead to neural dysfunction and symptomatology in schizophrenia. Dicarbonyl compounds can be effectively detoxified *via* the metabolic pathway of the glyoxalase system. MG reacts with glutathione (GSH) and is converted to S-D-lactoylglutathione (SLG) by Glo-1. SLG is catalyzed into D-lactate by glyoxalase-2 (Glo-2), and GSH is recycled. As a rate-limiting enzyme in the glyoxalase system, Glo-1 plays a critical role in the detoxification of carbonyl stress in schizophrenia.

We demonstrated that a novel functional SNP (rs1781735) in the promoter of the Glo-1 gene is associated with the risk of schizophrenia, and a large proportion of individuals with schizophrenia are homozygous for the rs1781735 T allele. Next, we identified the T allele of rs1781735, which effectively reduced the transcription activation of Glo-1 and was associated with the reduced RNA expression and enzymatic activity in the antipsychotic-naïve first-episode patients with schizophrenia. Finally, the evidence not only from the variants in rs1718735 but also from the mRNA expression and protein function suggested that Glo-1 is involved in the pathogenesis of schizophrenia in our Chinese cohorts (the hypothesized metabolic process is illustrated in Figure 4). To our knowledge, this is the first study to report a relationship between the rs1781735 and schizophrenia in a Chinese population.

In schizophrenia, the deficits in neural function (Shenton et al., 2001) are well-established phenotypic measures, that are related to the disease severity and outcome (Lieberman et al., 2001; Wojtalik et al., 2017). Imaging genetics is an emerging field to identify the genetic sources of brain activity and functional connectivity *in vivo* in a non-invasive manner (Krajcovic et al., 2019). However, there is still a gap in the understanding of the underlying molecular mechanisms by which the gene acts on the neural function phenotypes due to the absence of gene expression information in the corresponding and spontaneous brain regions. The eQTL could be an effective approach to bridge this gap by offering the genetic variations as a proxy to interpret gene expression levels in the brains (Battle et al., 2017; Huckins et al., 2019). It is necessary to combine eQTL analysis with imaging genetics research to elucidate the neural function phenotypes and the underlying molecular mechanisms.

Based on the evidence that Glo-1 is broadly expressed in the brains and that rs1718735 could be an eQTL for Glo-1, a neuroimaging analysis was further performed to investigate the relationship of Glo-1 and brain function in schizophrenia using rs1781735 as a proxy of Glo-1 expression. The rs1781735 TT genotype, which is associated with a low Glo-1 expression in brains, displayed decreased ALFF in the left middle frontal gyrus compared with the G carriers in the patients with schizophrenia. We can infer that the low expression of Glo-1 implies the decreased neural activity in the left middle frontal gyrus in patients with schizophrenia. This is in accordance with the brain expression pattern results showing that the highest Glo-1 expression in the frontal cortex (BA9) in GTEx (the brain areas involved but not completely overlapping with the neuroimaging atlas). The Gray matter reduction and dysfunction of the left middle frontal gyrus have been identified in schizophrenia (Li et al., 2018) and are associated with the symptoms of schizophrenia, such as delusion and language processing (Gao et al., 2015; Cantisani et al., 2018). Toriumi et al. reported that the combination of Glo-1 dysfunction and vitamin B6 (VB6) deficiency may cause behavioral deficits *via* mitochondrial dysfunction and oxidative stress in the frontal cortex in the schizophrenia mouse model (Toriumi et al., 2021). In this study, we first identified evidence that Glo-1 is associated with dysfunction in the frontal cortex in patients with schizophrenia. The combination of the evidence and our results introduce eQTL data as a bridge for the genetic imaging studies and provide a glimpse of the functional localization of Glo-1 in the brain.

The ethnic heterogeneity in the genetic cohort studies should be highlighted in this study. To date, the research on the relationship between the polymorphisms in the Glo-1 gene and schizophrenia susceptibility has been reported only in a Japanese population (Arai et al., 2010; Bangel et al., 2015). Arai found that a missense mutation polymorphism at rs4746 (Ala111Glu) and two heterozygous frameshift mutations were associated with the risk of schizophrenia, as demonstrated by DNA sequencing of 1,761 patients with schizophrenia and 1,921 control subjects. In the present study, we attempted to replicate the positive association between the rs4746 of the Glo-1 gene and schizophrenia, as reported by Arai et al. (2010), in our Han population of China, we did not find an association of rs4746 with schizophrenia. The same situation was observed for rs1781735, which has been reported to have a negative association with schizophrenia in the population of Japan. This discordance may stem from the ethnic differences that exist between the population of China and Japan. The MAF of rs4746 exhibits high population diversity: 0.354–0.475 in Europeans, 0.267 in sub-Saharan Africans, 0.239–0.395 in African Americans, and 0.033–0.125 in Asian populations (Junaid et al., 2004; Sacco et al., 2007; Wu et al., 2008). We detected 50 CC genotype carriers among the 2,092 Chinese individuals. The C allele frequency in the control group of the present study was 0.134, similar to the values in the Han Chinese population of Taiwan (the frequency for the controls was 0.124) and the HapMap database for the Han population of China, but the allele frequency was much lower in the population of Japan (0.033). For rs1781735, the frequency of the G allele in our cohort from China was 0.392, but in the cohort from Japan, it was 0.443. In addition, the diagnostic heterogeneity, cohort homogeneity, environmental exposure, sample sizes, and research strategies between the two studies may also explain these differences. Hence, the results of the genetic association studies need to be determined in other cohorts from different ethnic backgrounds.

There are possible limitations to this study. For the rs1781735 effect on mRNA expression and enzymatic activity analysis, the sample size in the rs1781735 GG sub-group is limited, especially in the control group, which may induce variation and unstable results (Supplementary Figure 4). But the result was supported by further analysis. For the mRNA expression analysis, the evidence was supported by eQTL analysis with 670 whole blood samples (Figure 2D). For the enzymatic activity, a significant result was observed in the GG+GT subgroup comparing with the TT subgroup. However, this issue still needs to be identified in a larger sample and the other cohorts. In the rs1781735 effect on the phenotypes of schizophrenia, we only perform the resting-state fMRI analysis. More comprehensive analysis of the neural characteristics, such as the brain structure, function connectivity analysis, and the relationship with the clinical characteristics, need to be investigated in further studies.

## Conclusions

To our knowledge, this study is the first to investigate Glo-1 risk in schizophrenia at multiple levels, such as genetic variants, transcription, expression, protein function, and phenotypes. Significant differences in variant distribution, RNA expression, and enzymatic activity were found between the patients with schizophrenia and controls. We first reported the effect of the Glo-1 gene on the neural function of schizophrenia using a functional SNP (rs1781735) as the proxy. The most prominent Glo-1 signal intensities were observed in the left middle frontal gyrus, suggesting that Glo-1 is involved in the dysfunction of the left middle frontal gyrus in schizophrenia. Glo-1 protects the nervous system against toxic carbonyl stress, and its reduced expression may play a key role in the etiology of schizophrenia. Our investigation lays the foundation for a better understanding of the influence of Glo-1 in schizophrenia and provides a referable analysis workflow in imaging genetics research.

## Data Availability Statement

The original contributions presented in the study are included in the article/Supplementary Material, further inquiries can be directed to the corresponding authors.

## Ethics Statement

The studies involving human participants were reviewed and approved by the Ethics Committee of Affiliated Hospital of Guangdong Medical University. The patients/participants provided their written informed consent to participate in this study. Written informed consent was obtained from the individual(s) for the publication of any potentially identifiable images or data included in this article.

## Author Contributions

GM, YW, KL, ZBL, WC, and YL conceived and designed the experiments. JY, CL, XZ, XX, QC, and XW performed the experiments. JY, SL, XL, BH, SX, DZ, ZT, JF, DL, ZD, XY, and ZXL collected the clinical samples and analyzed the data. JY and SL analyzed the data. JY and GM wrote the original draft. All authors contributed to the article and approved the submitted version.

## Funding

This study was supported by the National Natural Science Foundation of China (81670252, 81770034, and 81571157) and the Guangdong Basic and Applied Basic Foundation (2019A1515011306 and 2019A1515011424).

## Conflict of Interest

The authors declare that the research was conducted in the absence of any commercial or financial relationships that could be construed as a potential conflict of interest.

## Publisher's Note

All claims expressed in this article are solely those of the authors and do not necessarily represent those of their affiliated organizations, or those of the publisher, the editors and the reviewers. Any product that may be evaluated in this article, or claim that may be made by its manufacturer, is not guaranteed or endorsed by the publisher.
