# Supplementary material for: Glyoxalase 1 Confers Susceptibility to Schizophrenia: From Genetic Variants to Phenotypes of Neural Function
Source: Front Mol Neurosci. 2021 Nov 1;14:739526. doi: 10.3389/fnmol.2021.739526 (PMC8592033; doi:10.3389/fnmol.2021.739526)
Supplement: Supplementary file 1 [file Table_1.pdf]

Supplementary Material

Supplementary Tables

Table S1. The primers for real-time polymerase chain reaction

Gene	Purpose	Sequence (5'-3')	Tm (°C)
Glo-1	Forward	CCGCCATGATTCACATTTGA	55.75
	Revered	GTTGGCATGGCCTTTCCA	57.30
GADPH	Forward	ACCCACTCCTCCACCTTTGA	59.85
	Revered	CTGTTGCTGTAGCCAAATTCGT	58.21

Table S2. SNP information and oligonucleotide sequences used for Glo- 1 genotyping

SNP	Purpose		Sequence (5'→3')
rs4746	PCR primers	Forward	AGGGTCTGAATTGCCATTGTGGT
		Revered	TTTGTAGCAGGGGTTAGGCCAAT
	Primer extension		TTTTTTTTTTTTTTTTTTTTTTGGGGCACTGAAGATGATG
rs1781735	PCR primers	Forward	CACCAAGATGTCCCCGTCTCTC
		Revered	AATTTCTAGTCTTATGGAGCTGGTGTCTG
	Primer extension		TTTTTTTTTTTTTTTTTTTTTTTTTTTTTTTTTTTTTGGTGTCTGACAAACTGAAGCTG
rs1130534	PCR primers	Forward	GAGACTTAAAAGTAAATCATGGTGAGATGG
		Revered	GGACATTGTAGCTATGTTTTCCTTCC
	Primer extension		TTTTTTTTTTTTTTTTTTTTTTTTTTTTTTTTTTTTTTTTTTTTTGGCAATTCAGACCCTCGAGG
rs9470916	PCR primers	Forward	ACTCCAGCCCAAGAGCCAAGAG
		Revered	AAGGGGAGGAGGAAGGAAATGA
	Primer extension		TTTTTTTTTTTTTTTTTTTTTTTTTTTTTTTTTTTTTTTTTTTTTTTTTTTTCCTAGCCCAGTGTCTGGGTAC

Table S3. The primers for promoter luciferase activity assay

Gene	Purpose	Sequence (5'-3')	Enzyme
Glo-1	3'UTR-F	cacaactcgagTGCTGTGAGAATTCTCCTTTGAG	*Xho* I
	3'UTR-R	tgaggatccCGGAGGAGTCACCCACACTAC	*Bam*H I

Table S4. Analysis of associations of mRNA expression and enzymatic activity of Glo-1

	No.	Age (year)	RNA expression/Enzyme activity
**RNA expression analysis**			
**Total**			
Schizophrenia	63	31.25 ± 10.08	2.87 ± 1.58
Control	53	32.94 ± 9.04	5.68 ± 4.40
P		t = 0.94, *P* = 0.34	t = 4.42, $P = 3.98 \times 10^{-5}$
**Schizophrenia**			
GG	12	27.17 ± 5.20	3.73 ± 0.23
GT	24	32.91 ± 10.78	2.91 ± 2.08
TT	27	31.59 ± 10.84	2.46 ± 1.23
$P^{GG\ vs.\ TT}$		t = 1.34, *P* = 0.189	t = 3.54, *P* = 0.001
**Control**			
GG	10	34.16 ± 7.55	7.77 ± 5.33
GT	23	33.30 ± 9.62	5.61 ± 4.90
TT	20	32.5±9.51	4.23 ± 2.41
$P^{GG\ vs.\ TT}$		t =1.45 *P* = 0.885	t = 3.34, *P* = 0.003
**Enzyme activity analysis**			
**Total**			
Schizophrenia	74	29 ± 10.27	4.30 ± 1.94
Control	73	28.65 ±5.54	6.31 ± 2.83
P		t = 0.25, *P* = 0.802	t = 5.02, $P = 1.40 \times 10^{-6}$
**Schizophrenia**			
GG	15	29.6 ± 10.45	4.97 ± 1.39
GT	32	30.84 ± 11.13	4.51 ± 2.25
TT	27	26.48 ± 8.87	3.68 ± 1.67
$P^{GG\ vs.\ TT}$		t = 1.02, *P* = 0.312	t = 2.54, *P* = 0.015
$P^{GG + GT\ vs.\ TT}$		t = 1.62, *P* = 0.111	t = 2.13, *P* = 0.036
**Control**			
GG	7	30.57 ± 5.26	8.01 ± 5.60
GT	36	28.27 ± 5.60	6.68 ± 5.61
TT	30	28.66 ± 5.61	5.48 ± 5.54
$P^{GG\ vs.\ TT}$		t = 0.82, *P* = 0.419	t = 2.59, *P*=0.014
$P^{GG + GT\ vs.\ TT}$		t = 0.01, *P* = 0.991	t = 2.16, *P* = 0.034

Abbreviations: No: number; *:$P < 0.05$.

Table S5. Demographic characteristics of sample for analysis of genotype and allele distributions

Variables		Patients	Controls	Statistical tests
Total Number		1069	1023	
Mean Age ± SD (year)		34.42 ±13.15	34.16 ± 9.35	t=0.51 *P*=0.61
Gender N (%)	Male	684 (63.9%)	619(60.5%)	$\chi2$=2.69
	Female	385(36.1%)	404(39.5%)	*P*=0.10
Age at onset (Mean ± SD, year)		24.96 ± 9.51		
Duration (Mean ± SD, month)		125.82 ± 185.49		
Family history: n (%)				
+		139		
-		930		
PANSS (Mean ± SD, year)				
total score		77.34 ± 19.24		
P subscore		21.60 ± 7.49		
N subscore		18.0 3± 8.78		
G subscore		36.35 ± 9.64		

Abbreviations: SD: standard deviation; N: number; PANSS: Positive and Negative Syndrome Scale; P: Positive scale; N: Negative scale; G: General Psychopathology scale.

Table S6. Estimation of LD between each pair of loci in Glo-1

SNP	rs1130534		rs4746		rs1781735	
	D'	R^2	D'	R^2	D'	R^2
rs9470916	0.972	0.924	1.0	0.059	0.967	0.188
rs1130534	—	—	1.0	0.061	0.995	0.204
rs4746	—	—	—	—	0.934	0.085

Abbreviations: LD: Linkage disequilibrium

Table S7. Haplotype analysis of SNPs in the Glo-1 gene in schizophrenic patients and controls

rs9470916 – rs1130534 – rs4746- rs1781735	Frequency	Schizophrenia		Controls		$\chi2$	*P*	OR (95 % CI)
		N	%	N	%			
C-T-A-G	0.356	660.6	33.84	666.4	37.44	5.13	0.024*	0.86(0.75 - 0.98)
A-A-A-T	0.255	510.9	26.17	438.9	24.66	1.13	0.288	1.08(0.94 - 1.26)
C-T-A-T	0.23	455.3	23.32	404.5	22.73	0.17	0.680	1.03(0.89 - 1.20)
C-T-C-T	0.14	285.1	14.61	235.9	13.25	1.40	0.237	1.12(0.93 - 1.35)

Haplotype frequency <0.03 in both schizophrenic patients and controls has been dropped.
Abbreviations: N: number; OR: odds ratio; 95% CI 95% confidence interval.

Table S8. The POWER calculation for analysis of allele and genotype distribution of Glo-1 polymorphisms

SNP	Case	Control	MAF	Power
rs1781735	1069	1023	0.41	1
rs9470916	1069	1023	0.2	1
rs1130534	1069	1023	0.31	1
rs4746	1069	1023	0.11	0.993

Abbreviations: MAF: minor allele frequency. Power was calculated using the MAF of each allele and with an OR of 1.5 at the 0.05 level.

Table S9. Demographic and clinical characteristics for schizophrenia patients and healthy controls in the imaging-genetics subgroup

	TT	G carriers	Statistical tests	*P*
	n=40	n=51		
Age (Mean ± SD, years)	26.77±7.38	29.07±8.36	t = 1.21	0.23
Sex, NO. Male/Female	23/17	30/21	χ2=0.02	1.00
Years of education (Mean ± SD, years)	10.73±2.61	10.56±3.32	t =0.24	0.81
Age of onset (Mean ± SD, years)	25.76±7.51	27.76±9.05	t=0.97	0.33
Duration of psychosis (Mean ± SD, months)	13.29±22.06	6.56 ±14.01	t=1.56	0.12
PANSS (Mean ± SD)				
Total scores of PANSS	75.93±22.23	76.83±18.64	t=0.18	0.86
Positive scores of PANSS	23.69±9.52	24.22±7.74	t=0.26	0.80
Negative scores of PANSS	16.69±12.88	17.78±12.48	t=0.36	0.72
General psychopathological scores of PANSS	35.55±10.52	34.83±9.22	t=0.30	0.76
Family psychotic history, NO. PFH/NFH	3/36	7/44	χ2=0.81	0.51

Abbreviations: NFH, negative family history; PFH, positive family history; PANSS, Positive and Negative Syndrome Scale.
